# Discovery and genetic characterization of novel paramyxoviruses from small mammals in Hubei Province, Central China

**DOI:** 10.1099/mgen.0.001229

**Published:** 2024-05-03

**Authors:** Jia-le Xu, Jin-tao Chen, Bing Hu, Wei-wei Guo, Jing-jing Guo, Chao-rui Xiong, Ling-xin Qin, Xin-nai Yu, Xiao-min Chen, Kun Cai, Yi-rong Li, Man-qing Liu, Liang-jun Chen, Wei Hou

**Affiliations:** 1 State Key Laboratory of Virology/Department of Laboratory Medicine/Hubei Provincial Key Laboratory of Allergy and Immunology, School of Basic Medical Sciences/Zhongnan Hospital, Wuhan University, 185 Donghu Road, Wuhan, Hubei, 430071, PR China; 2 Institute of Health Inspection and Testing, Hubei Provincial Center for Disease Control & Prevention, 6 Zhuodaoquan Road, Wuhan, Hubei, 430079, PR China; 3 Division of Virology, Wuhan Center for Disease Control & Prevention, 288 Machang Road, Wuhan, Hubei, 430015, PR China; 4 School of Public Health, Wuhan University, 185 Donghu Road, Wuhan, Hubei, 430071, PR China

**Keywords:** co-divergence, diversity, genome organization, *Jeilongvirus*, Paramyxovirus

## Abstract

Paramyxoviruses are a group of single-stranded, negative-sense RNA viruses, some of which are responsible for acute human disease, including parainfluenza virus, measles virus, Nipah virus and Hendra virus. In recent years, a large number of novel paramyxoviruses, particularly members of the genus *Jeilongvirus*, have been discovered in wild mammals, suggesting that the diversity of paramyxoviruses may be underestimated. Here we used hemi-nested reverse transcription PCR to obtain 190 paramyxovirus sequences from 969 small mammals in Hubei Province, Central China. These newly identified paramyxoviruses were classified into four clades: genera *Jeilongvirus*, *Morbillivirus*, *Henipavirus* and *Narmovirus*, with most of them belonging to the genus *Jeilongvirus*. Using Illumina sequencing and Sanger sequencing, we successfully recovered six near-full-length genomes with different genomic organizations, revealing the more complex genome content of paramyxoviruses. Co-divergence analysis of jeilongviruses and their known hosts indicates that host-switching occurred more frequently in the evolutionary histories of the genus *Jeilongvirus*. Together, our findings demonstrate the high prevalence of paramyxoviruses in small mammals, especially jeilongviruses, and highlight the diversity of paramyxoviruses and their genome content, as well as the evolution of jeilongviruses.

## Data Summary

The high-throughput sequencing reads generated in this study have been deposited in the SRA database under accession code PRJNA1088191. All sequences generated in this study have been deposited with GenBank under accession numbers: OQ943404 to OQ943593 for partial sequences of the polymerase gene and OQ970174 to OQ970179 for near full-length genomes.

**Impact Statement**

The family *Paramyxoviridae* contains several zoonotic viruses, such as Nipah virus and Hendra virus. Mammals, particularly rodents, have been identified as natural reservoirs for many zoonotic viruses that can cause severe disease in humans once they cross species barriers. In recent years, a large number of novel paramyxoviruses have been discovered in wild mammals, such as Langya henipavirus, which was pathogenic to humans. Therefore, it is important to investigate viral diversity for estimating the potential risk of the emergence of novel human pathogens from the family *Paramyxoviridae*. This study demonstrates the high prevalence and diversity of paramyxoviruses in small mammals, especially jeilongviruses. In addition to Beilong virus, the near-full-length genomes of five novel paramyxoviruses with different genomic organizations, including three jeilongviruses, one henipavirus and one morbilli-like virus, were characterized, revealing the more complex genome content of paramyxoviruses. The findings also suggest more frequent host-switching events in jeilongviruses in its rodent reservoir, which may lead to the rapid expansion of jeilongviruses.

## Introduction

The emergence and re-emergence of infectious diseases, particularly zoonoses, pose a significant threat to public health and socio-economic stability worldwide [1,3]. Wildlife plays a crucial role in the emergence of zoonotic diseases by providing a ‘zoonotic pool’ [4]. Notably, most zoonotic viruses described to date are hosted in mammals, particularly rodents, which carry at least 85 unique zoonotic diseases [5,6]. In recent years, outbreaks of zoonotic viruses such as severe acute respiratory syndrome coronavirus 2 (SARS-CoV-2), SARS-CoV, Middle East respiratory syndrome coronavirus (MERS-CoV), Nipah virus and Hendra virus have caused severe diseases in humans, emphasizing the importance of investigating viral evolution and diversity in wildlife sources [7,11]. Therefore, continuous surveillance of potential viruses from wildlife species is crucial for preventing and mitigating outbreaks of emerging and re-emerging zoonoses.

Paramyxoviruses are a large diverse group of non-segmented, negative-sense ssRNA viruses that can infect a wide range of hosts, from reptiles, birds and fishes to a variety of mammals [12]. Paramyxoviruses include several important animal pathogens, such as rinderpest virus and peste des petits ruminants virus, which cause highly contagious transboundary animal disease with severe socio-economic impact on the livestock industry [13,14]. Additionally, some paramyxoviruses can also cause diseases in humans, including measles virus, mumps virus and parainfluenza virus. Highly pathogenic zoonotic paramyxoviruses, such as Hendra virus and Nipah virus, also pose a significant public health threat [12,15, 16]. Currently, the family *Paramyxoviridae* comprises four subfamilies, 17 genera and 86 species (https://talk.ictvonline.org/, accessed 4 March 2023), and virus genomes range from 12 496 to 20 148 nt [17]. All paramyxoviruses contain six core genes, encoding the viral nucleoprotein (N), phosphoprotein (P), matrix protein (M), fusion protein (F), receptor binding protein (RBP) and RNA polymerase (L). It is worth noting that some paramyxoviruses, including members of the newly established (in 2020) genus *Jeilongvirus*, have one or two additional genes that encode accessory proteins, such as small hydrophobic protein (SH) and transmembrane protein (TM), adding complexity to the genomic content of paramyxovirus [17].

Recently, due to improvements in sequencing technologies, a large number of novel paramyxoviruses were discovered in various hosts around the world, indicating that the true diversity of the family *Paramyxoviridae* remains to be determined [18,25]. Notably, most of these novel paramyxoviruses were considered new members of the genus *Jeilongvirus*, and while rats and bats are the main reservoir hosts of jeilongviruses, the identification of novel jeilongviruses in non-rat/non-bat hosts suggests the possibility of cross-species transmission events [20,26, 27]. Although it is currently unknown whether jeilongviruses can infect humans and cause human disease, it is important to note that other paramyxovirus genera, such as *Henipavirus* and *Morbillivirus*, which are known to cause diseases in both animals and humans, have also been observed to spillover from their natural hosts [26,28]. For example, Langya henipavirus, which is thought to be carried by shrews, has recently emerged and caused disease in humans [28]. Therefore, the increasing diversity of paramyxoviruses and the expansion of their hosts pose a potential risk for the emergence of novel human pathogens that cannot be ignored.

In this study, 969 small mammals, including 964 rodents and five insectivores, were captured from six cities in Hubei Province and screened for the presence of paramyxoviruses using hemi-nested reverse transcription PCR (RT-PCR) to gain a better understanding of the epidemiology and diversity of paramyxoviruses in Hubei Province. Additionally, the genomic structures of five novel paramyxoviruses, including three jeilongviruses, one henipavirus and one morbilli-like virus, were characterized. To gain insights into the evolution of jeilongviruses, we also investigated the frequency of cross-species transmission.

## Methods

### Trapping of small mammals and sample collection

**Fig. 1.**
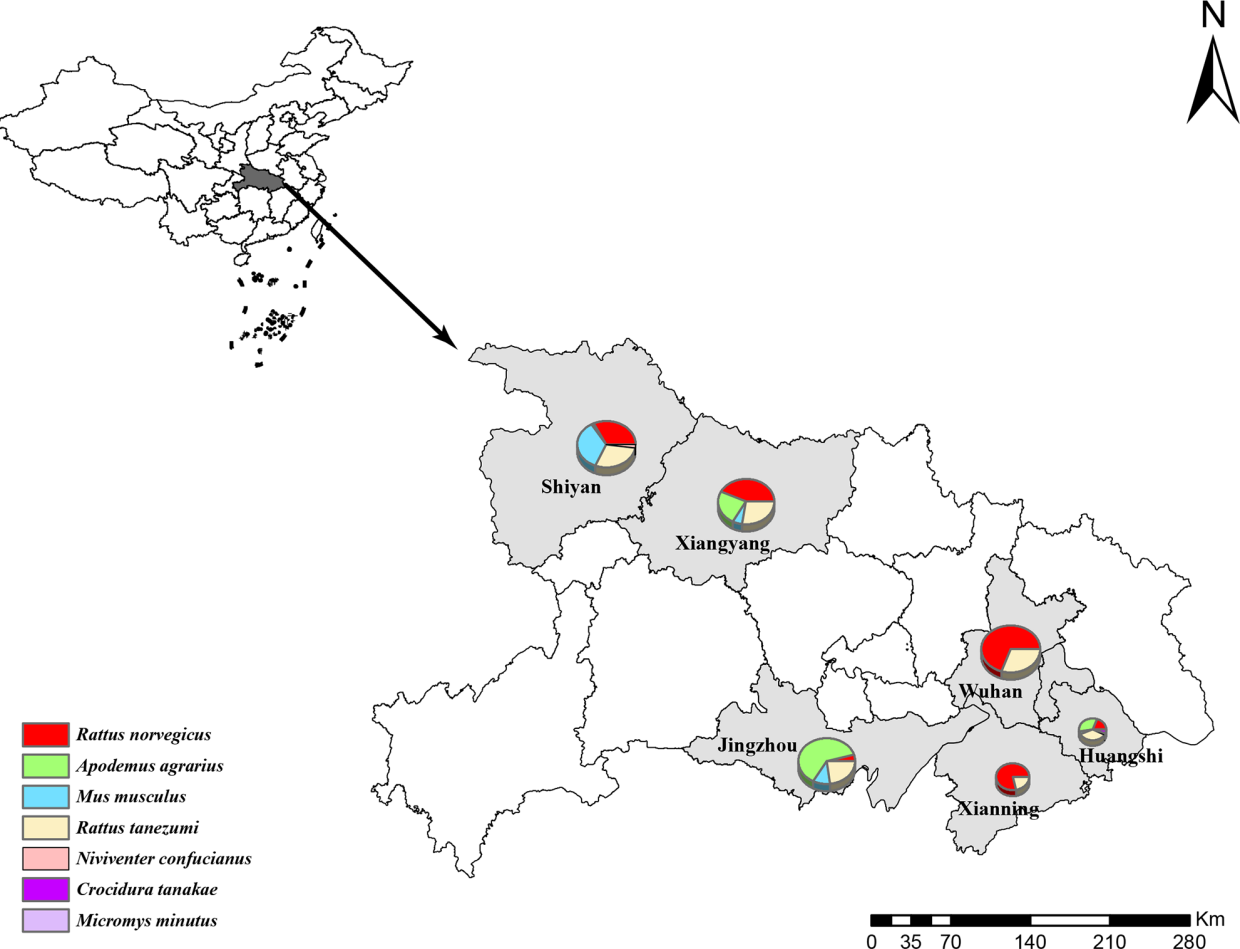
Geographical map showing the collection sites for small mammals. The sampling cities in this study are indicated in grey. Pie chart size is according to the numbers of samples collected. The composition of the sample species is shown by different colours in the pie chart. This map was created in ArcGIS 10.8 software (ESRI) and Adobe Illustrator vCC2018 (Adobe).

Wild small mammals, including rodents and insectivores, were captured with cage traps deployed with food bait in the cities of Wuhan, Shiyan, Huangshi, Jingzhou, Xiangyang and Xianning, Hubei Province, in 2021 (Fig. 1). All sampling work was carried out by the Hubei Provincal Center for Disease Control and Prevention as part of the surveillance of zoonotic diseases. Based on humanitarian considerations, the animals were captured alive and euthanized by isoflurane inhalation before dissection, and every effort was made to minimize suffering. The species of animals were identified using morphological examination and further confirmed by sequence analysis of the mitochondrial (mt)-*cyt* b gene [29,30]. Previous studies have found that the highest viral loads and prevalence rate of paramyxovirus in rodents were seen in the kidneys [26,31]. Therefore, we collected kidney tissue samples from rodents and insectivores and stored them at −80 °C until further use in this study. All animal experiments were conducted in accordance with the internationally accepted principles and guidelines for the Care and Use of Laboratory Animals of Wuhan University and approved by the ethics committee of Wuhan University.

### DNA and RNA extraction, paramyxovirus detection and full genome sequencing

Both DNA and RNA were simultaneously extracted from kidney tissue using a DNA/RNA extraction kit (Omega) according to protocols suggested by the manufacturer. The mt-*cyt* b gene (1140 bp) was amplified by PCR with the one primer pair for rodents [29] and one for insectivores [30]. Paramyxovirus RNA was then detected by hemi-nested RT-PCR targeting a conserved region of the polymerase gene of paramyxoviruses from the subfamily *Orthoparamyxovirinae* [32].

For the novel paramyxoviruses identified in this study, we used high-throughput sequencing (HTS) and RT-PCR to obtain whole genome information (Table S1, available in the online version of this article). RNA sequencing library construction and rRNA depletion were performed using the Zymo-Seq RiboFree Total RNA Library Kit (Zymo Research). Then, paired-end (150 bp) sequencing of the four dual-indexed libraries was performed using the Illumina NovaSeq 6000 platform. A brief description of the pipeline for assembly and annotation of the newly identified paramyxovirus genome was as follows. First, adaptor- and quality-filtered sequencing reads were assembled *de novo* using MEGAHIT (v1.2.8). Viral contigs were identified by BLASTX search (using Diamond v0.9.25 [33]) against the NCBI non-redundant database (downloaded 10 March 2023) with e-values set to 0.001.We attempted to determine the complete genome of the viruses using rapid amplification of cDNA ends but were unable to successfully amplify the terminal 5′ and 3′ ends due to limited template amounts or degradation.

PCR amplicon products with the expected size were separated by agarose gel electrophoresis and purified using a gel extraction kit (TaKaRa) according to the manufacturer’s recommendations. PCR amplicons (<700 bp) were subjected to direct Sanger sequencing, while those >700 bp were cloned and inserted into the pMD18-T vector (Takara) and then transformed into JM109-143 competent cells, and at least three positive clones were selected for sequencing.

### Phylogenetic analysis

The partial sequences (407 bp) of a conserved region in the polymerase gene of paramyxoviruses obtained in this study and the reference sequences downloaded from GenBank were aligned by the ClustalW method in MEGA-X [34]. Phylogenetic trees of paramyxoviruses were reconstructed using the maximum likelihood (ML) method implemented in IQ-TREE v2.0 [35] with 1000 bootstrap replicates, employing the best-fit evolutionary model TIM2+F+I+G4.

For near-complete genomes of paramyxoviruses identified in this study, ORFs were predicted using the NCBI ORF finder (ncbi.nlm.nih.gov/orffinder). Alignments for each ORF of paramyxoviruses from this study and representative members of the subfamily *Orthoparamyxovirinae* were made by MAFFT v7 and then trimmed using Gblocks v0.91b to remove gaps and highly variable regions [36,37]. Similarly, the ML phylogenetic trees of N, P, M, F, RBP and L genes were made in IQ-TREE v2.0, employing a GTR+F+I+G4 nucleotide substitution model, with 1000 bootstrap replicates. Multiple alignments of G protein sequences of jeilongviruses were conducted by DNAMAN v5.2.9.

All resulting trees were exported to the Interactive Tree of Life (iTOL) (http://itol.embl.de, accessed 9 January 2023) for visualization, modification and annotation [38].

### Co-divergence analysis

To investigate the co-phylogenetic patterns between jeilongviruses and their hosts, we performed event-based co-phylogenetic reconstructions in the Jane package v4 to estimate the relative frequencies of co-divergence and cross-species transmission in the evolutionary histories of the genus *Jeilongvirus* [39]. RNA polymerase gene sequences from members of the genus *Jeilongvirus* in NCBI Virus (https://www.ncbi.nlm.nih.gov/labs/virus, accessed 12 December 2022), including unclassified jeilongviruses, were downloaded, along with the *cyt* b gene sequences of their hosts from GenBank. Then, the sequences of viruses and hosts were aligned using MAFFT v7. Before performing the reconstructions, the complexity of the jeilongvirus and host phylogenies was reduced as much as possible. ML phylogenetic trees of the hosts and virus were reconstructed using IQ-TREE v2.0, with the GTR+F+I+G4 nucleotide substitution model and 1000 bootstrap replicates.

Jane was used to perform mapping of five virus phylogeny events (co-speciation, duplication, duplication with host-switching, loss, and failure to diverge) onto the host tree, with each event assigned to a cost. A best mapping was sought by minimizing the total cost. The event costs were set as previously described: 0 for co-divergence, 1 for duplication, 1 for duplication with host-switching, 1 for loss and 1 for failure to diverge [40], and 0 for co-divergence, 1 for duplication, 2 for host-switching, 1 for loss and 1 for failure to diverge [41]. Statistical analyses were performed using the random parasite tree method with a sample size of 500. Finally, the co-divergence pattern was visualized using TreeMap [42], and the untangle function was used to minimize the number of crossed lines.

### Statistical analysis

Statistical analysis was performed using the Statistical Package for Social Sciences v27.0 software (SPSS) to test whether the positivity rate varied by host species and sampling site (*P*<0.05).

### Recombination analysis

To assess putative recombination events, the full genome alignments of all rodent paramyxoviruses were scanned using seven recombination detection methods (RDP, GENECONV, Bootscan, Maxchi, Chimaera, Siscan and 3Seq) within the Recombination Detection Program v4.101 (RDP4) package [43]. A confirmed recombination event was only considered when the event could be verified by four or more methods with *P*<0.05.

## Results

### Sample collection and paramyxovirus screening

In 2021, a total of 969 small mammals were captured from six cities in Hubei Province: Jingzhou, Xiangyang, Shiyan, Huangshi, Xianning and Wuhan. The captured animals included 964 rodents from six different species: 389 *Rattus norvegicus*, 192 *Apodemus agrarius*, 107 *Mus musculus*, 270 *Rattus tanezumi*, five *Niviventer confucianus* and one *Micromys minutus*. Additionally, five *Crocidura tanakae* insectivores were captured. The species, number and geographical distribution of these samples are shown in Table 1.

**Table:** 

Species	Jingzhou (%)	Xiangyang (%)	Shiyan (%)	Huangshi (%)	Xianning (%)	Wuhan (%)	Total (%)
*Rattus norvegicus*	)	33/87 (37.9)	20/70 (28.6)	2/11 (1.8)	27/63 (42.9)	25/150 (16.7)	111/389 (28.5)
*Apodemus agrarius*	47/125 (37.6)	)	1/3 (33.3)	0/19 (0)	0 (0)	0 (0)	57/192 (29.7)
*Mus musculus*	4/23 (17.4)	3/13 (23.1)	1/69 (1.4)	0/2 (0)	0 (0)	0 (0)	8/107 (7.5)
*Rattus tanezumi*	2/46 (4.3)	1/55 (1.8)	1/64 (1.6)	1/21 (4.8)	0/17 (0)	3/67 (4.5)	8/270 (3.9)
*Niviventer confucianus*	0 (0)	0 (0)	2/4 (50.0)	1/1 (100.0)	0 (0)	0 (0)	3/5 (60.0)
*Micromys minutus*	1/1 (100.0)	0 (0)	0 (0)	0 (0)	0 (0)	0 (0)	1/1 (100.0)
*Crocidura tanakae*	0 (0)	0 (0)	2/2 (100.0)	0/3 (0)	0 (0)	0 (0)	2/5 (40.0)
Total	58/203 (28.6)	46/200 (23.0)	27/212 (12.7)	4/57 (7.0)	27/80 (33.8)	28/217 (12.9)	190/969 (19.6)

Of the 969 samples, 190 (19.6 %) tested positive for paramyxovirus RNA, with significant variation in positivity rates between host species and collection locations (*P*<0.001). All six rodent species tested positive for paramyxovirus RNA, with the highest rates observed in *A. agrarius* (29.7 %, 57/192) and *R. norvegicus* (28.5 %, 111/389). Geographically, the highest rates were observed in Xianning (33.8 %, 27/80), Jingzhou (28.6 %, 58/203) and Xiangyang (23.0 %, 46/200), while rates were lower in Wuhan (12.9 %, 28/217), Shiyan (12.7 %, 27/212) and Huangshi (7.0 %, 4/57). Paramyxovirus RNA was also detected in shrews (40 %, 2/5) in Shiyan city. In conclusion, our survey showed that paramyxovirus-positive samples were distributed across all six sampling sites and seven different hosts.

### Diversity of paramyxoviruses in Hubei Province

**Fig. 2.**
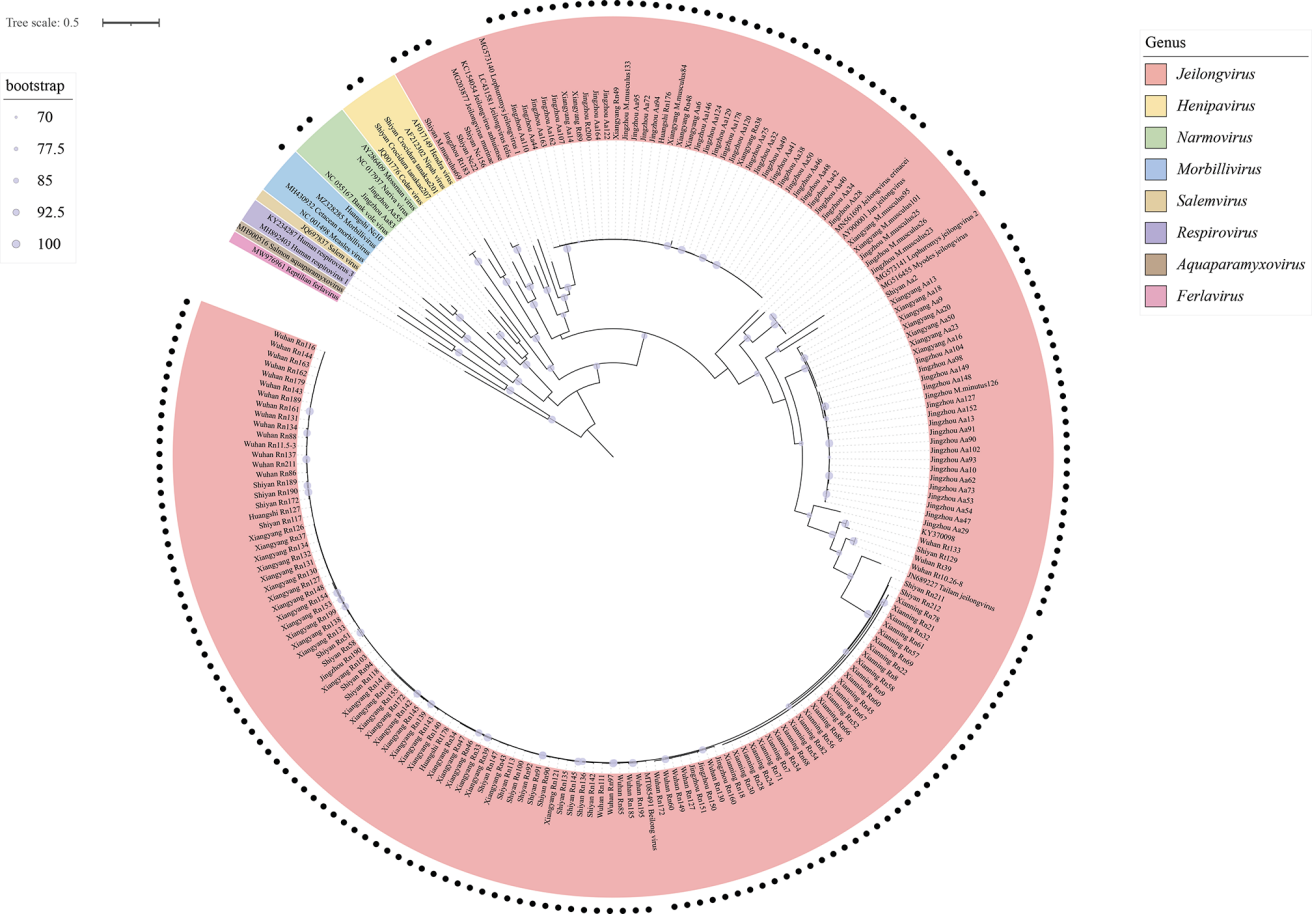
>

The 190 paramyxovirus sequences obtained in this study were divided into four clades based on the ML phylogenetic tree reconstructed using partial sequences of the paramyxovirus L gene (Fig. 2 and Table 2). Specifically, 185 paramyxoviruses from different murine species formed a well-supported clade (bootstrap=89) with other recognized jeilongviruses, while two paramyxoviruses from *A. agrarius* (Jingzhou55 and Jingzhou83) clustered within the genus *Narmovirus*, and another paramyxovirus from *Niviventer confucianus* (Huangshi10) was closely related to morbilliviruses. Interestingly, the paramyxoviruses from two *Crocidura tanakae* samples (Shiyan201 and Shiyan207) belonged to the genus *Henipavirus* (bootstrap=96).

**Table 2.** 

	*Jeilongvirus*		*Henipavirus* sp.	Morbilli-like virus	*Narmovirus* sp.	Total
	Beilong virus	Jeilongvirus sp.				
**Species**						
*Rattus norvegicus*	107	4	0	0	0	111
*Apodemus agrarius*	0	55	0	0	2	57
*Mus musculus*	0	8	0	0	0	8
*Rattus tanezumi*	1	7	0	0	0	8
*Niviventer confucianus*	0	2	0	1	0	3
*Crocidura tanakae*	0	0	2	0	0	2
*Micromys minutus*	0	1	0	0	0	1
**City**						
Jingzhou	4	52	0	0	2	58
Xiangyang	30	16	0	0	0	46
Shiyan	20	5	2	0	0	27
Huangshi	2	1	0	1	0	3
Xianning	27	0	0	0	0	27
Wuhan	25	3	0	0	0	28
Total	108	77	2	1	2	190

We found that the majority of the detected paramyxoviruses (97.4 %, 185/190) belonged to the genus *Jeilongvirus*, with 108 paramyxoviruses (58.4 %, 108/185) clustering with Beilong virus (bootstrap=100). Notably, almost all of these Beilong viruses were found in *R. norvegicus* (99.1 %, 107/108), which is a widely distributed species in Hubei Province and may have contributed to the wide distribution of Beilong virus across the six sampling sites (Table 2). In addition to Beilong virus, we also identified eight potential novel species within the genus *Jeilongvirus*, each forming an independent clade and showing a sister relationship with other recognized species. Similarly, based on phylogenetic position in the ML tree reconstructed using the partial L gene sequences, the remaining paramyxoviruses identified in this study may represent novel species of morbillivirus, narmovirus and henipavirus. Strikingly, the genus *Jeilongvirus* described here comprised viruses identified in all six rodent species at six investigation sites. Overall, these findings indicate the extensive diversity and widespread geographical distribution of paramyxoviruses, represented by jeilongviruses, in Hubei Province.

### Phylogenetic analysis among newly identified and known paramyxoviruses

To better characterize the newly identified paramyxoviruses, HTS and RT-PCR were used to obtain more sequence information for representative samples. The scaffolds identified through HTS analysis included those from Jingzhou151, Jingzhou25, Jingzhou148 and Shiyan22 samples. Specifically, there were 947, 3432, 1914 and 16 589 reads corresponding to the genomes of these samples, respectively, with average sequencing depths of 7.23, 26.83, 14.22 and 132.60. The genome coverage achieved was 47.50, 90.78, 83.54 and 53.38 %, respectively. To address the gapped regions and low-abundance areas identified in the HTS, we performed additional verification using RT-PCR and Sanger sequencing methods. We successfully recovered the complete coding regions of six representative samples from different branches in the ML phylogenetic tree reconstructed using partial sequences of the paramyxovirus L gene. We named these viruses as Beilong virus strain Rn/Jingzhou151 (Jingzhou151), Jingzhou Mus musculus jeilongvirus (Jingzhou25), Jingzhou Apodemus agrarius jeilongvirus (Jingzhou148), Shiyan Niviventer confucianus jeilongvirus (Shiyan22), Shiyan Crocidura tanakae henipavirus (Shiyan201) and Huangshi Niviventer confucianus morbilli-like virus (Huangshi10). These samples were analysed together with the Beilong virus sequences previously obtained by our research group (GenBank: MN598981–MN598984).

Fig. 3 % %

**Fig. 3.**
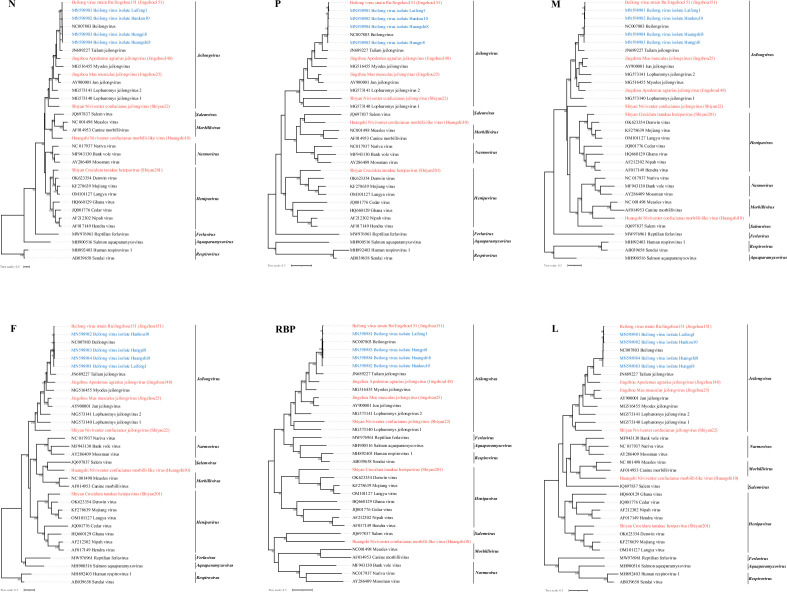


Besides jeilongviruses, we also found a novel morbilli-like virus (Huangshi10) from *Niviventer confucianus*. Huangshi10 was closely related to members of the genus *Morbillivirus* in all N, M and L gene trees. Additionally, in the P, F and RBP gene trees, Huangshi10 clustered with Salem virus and had a sister relationship with other morbilliviruses, with no significant evidence of recombination. Moreover, we identified a novel henipavirus (Shiyan201) from *Crocidura tanakae*, a species of shrew, which is divergent from the rodent-borne paramyxoviruses detected in this study. In all six gene trees, Shiyan201 formed a well-supported sister clade to henipaviruses and showed a closer evolutionary relationship with Mojiang virus (67.1 % nucleotide identity). These results show that paramyxoviruses identified in this study may represent novel members of the genera *Jeilongvirus* and *Henipavirus*, and a novel morbilli-like virus.

### Genomic characteristics

**Fig. 4.**
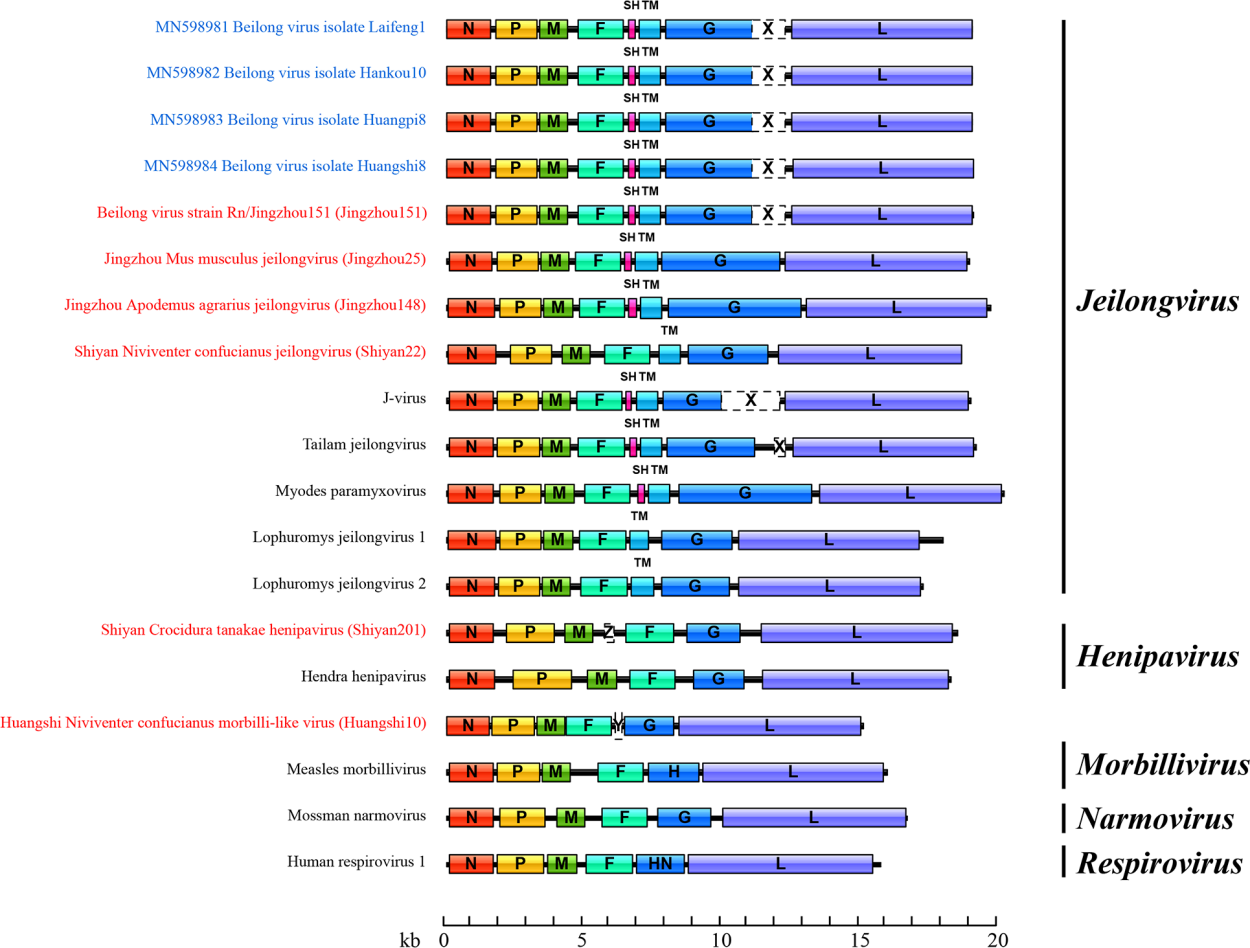
are

We attempted to determine the complete genome of the viruses using rapid amplification of cDNA ends but were unable to successfully amplify the terminal 5′ and 3′ ends due to limited template amounts or degradation. The ORFs of the near-full-length genome obtained in this study are depicted in Fig. 4. In the genus *Jeilongvirus*, Laifeng1, Hankou10, Huangpi8, Huangshi8, Jingzhou151, Jingzhou25 and Jingzhou148 were predicted to have two ORFs between the F and G genes, encoding SH and TM proteins, and a putative RNA editing site in their P gene was ‘TTAAAAAAGGCA’. In contrast, Shiyan22 encoded only the TM protein, and the RNA editing site in the P gene was ‘TTAAAAGGGGCA’, which distinguished it from the other jeilongviruses found in this study. In addition, in the genomes of Laifeng1, Hankou10, Huangpi8, Huangshi8 and Jingzhou151 (which appear to belong to Beilong virus), the G gene was followed by an ORF X of unknown function, consistent with the genomic characteristics of Beilong virus described in a previous study [44]. Interestingly, while Jingzhou25 was closely related to J-virus, ORF X was absent in its genome. The size of the G gene was also noteworthy. The G proteins of Jingzhou148, Jingzhou25 and Shiyan22 were 1602, 1412 and 959 aa long, respectively, in contrast to the typical 600 aa found in the G protein of paramyxoviruses [45]. Subsequently, we aligned the G protein sequences of these jeilongviruses. Interestingly, the first 600 aa of these G proteins showed a certain degree of conservation, indicating similarity. However, the remaining portions of the sequences showed little resemblance or conservation (Fig. S2).

For Huangshi10 and Shiyan201, the novel henipavirus in *Crocidura tanakae* and the novel morbilli-like virus in *Niviventer confucianus*, the predicted RNA editing sites were ‘TTAAAAAGGGCA’ and ‘TTAAAAAAGGCA’, respectively. These sequences, including the editing sites mentioned, matched a conserved motif sequence (YTAAAARRGGCA) found in all members of the genera *Morbillivirus* and *Henipavirus*, as well as in J-virus, Beilong virus, Tailam virus and rodent paramyxovirus. Additionally, we also identified an additional ORF Y and ORF Z in the genomes of Huangshi10 and Shiyan201, located between the F and G ORFs and the M and F ORFs, respectively, which is absent in other morbilliviruses and henipaviruses identified by ICTV. The prediction of transmembrane helices suggests that ORF Y and ORF Z encode small transmembrane proteins of 74 and 118 aa, respectively (Fig. S1). However, whether these two additional ORFs are biologically active remains to be determined.

### Co-phylogenetic relationships of jeilongviruses and their hosts

**Fig. 5.**
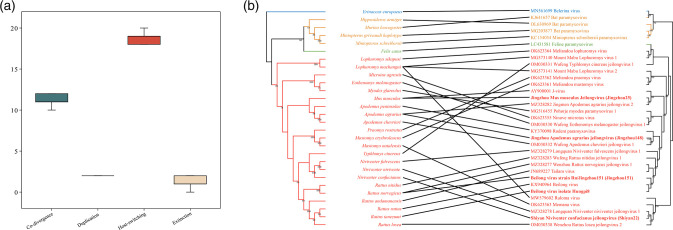


Since the establishment of the genus *Jeilongvirus*, many new paramyxoviruses from rodents, bats and even cats and hedgehogs have been classified in this genus. In this study, jeilongviruses also exhibited significant diversity, wide host range and complex genomic content. To better understand the evolutionary history of jeilongviruses, we compared the phylogenetic topologies of jeilongviruses and their hosts using an event-based method. The relative frequencies of the four evolutionary events were 10–12 for co-divergence events, 2 for lineage duplications, 18–20 for host switches and 0–2 for losses (Fig. 5a). The results showed a relatively high frequency of host-switching for jeilongviruses, which was also reflected in the virus–host tanglegrams (Fig. 5b). While the tree topologies of viruses and their hosts showed significant co-divergence at the level of host order, at the species level, we found clear inconsistencies between jeilongviruses and their rodent hosts. Collectively, these results suggest that there is no significant co-divergence relationship between jeilongviruses and their hosts (*P*>0.05), but instead, jeilongviruses undergo frequent cross-species transmission.

## Discussion

Rodents have been identified as natural reservoirs for many zoonotic viruses that can cause severe disease in humans once they cross species barriers [46]. The family *Paramyxoviridae* contains several zoonotic viruses, such as Nipah virus and Hendra virus. In the past decade, the widespread use of next-generation sequencing has led to the identification of a large number of novel paramyxoviruses with (near-) complete genome sequences that exhibit a wide host range and geographical distribution [19,23, 24, 47]. However, little was previously known about the diversity, geographical distribution and evolution of paramyxoviruses in Hubei Province. In this study, we investigated the prevalence of paramyxoviruses in 969 small mammals (964 rodents and five insectivores) from seven different species sampled in six cities in Hubei Province, with a strong focus on rodents. In total, we discovered 190 paramyxovirus-positive samples, with an overall positivity rate of 19.6 %. This percentage is largely consistent with the positive rate of paramyxovirus in small mammals reported in other studies [23,48,50]. Notably, the positive samples were distributed across all six sampling sites and seven different hosts, revealing the high prevalence and wide geographical distribution of paramyxoviruses in Hubei Province.

According to phylogenetic analysis, the 190 paramyxovirus sequences obtained in this study belong to four well-supported clades: the genera *Jeilongvirus*, *Morbillivirus*, *Henipavirus* and *Narmovirus*, revealing the broad diversity of paramyxovirus in small mammals in Hubei Province (Fig. 2 and Table 2). Additionally, we successfully determined six near-complete genomes using RT-PCR, Sanger sequencing and HTS, including four jeilongviruses, one henipavirus and one morbilli-like virus. The genus *Jeilongvirus* was established by the ICTV in 2019, including six species after a thorough assessment. However, the genus has rapidly expanded in recent years, with many unclassified jeilongviruses being discovered [25,47, 51, 52]. In this study, 185 paramyxovirus sequences (97.4 %, 185/190) were confirmed to cluster within the genus *Jeilongvirus*. Notably, most of these jeilongviruses are closely related to Beilong virus (58.4 %, 108/185), and the remaining jeilongviruses, distributed across all six rodent species identified in this study, appear to belong to eight potential novel species awaiting classification. Phylogenetic trees reconstructed based on six core genes indicated that the four jeilongviruses identified in this study fall into four different clades, exhibiting high genetic diversity. Notably, the Beilong virus strain Rn/Jingzhou151 exhibited 2.5 –3.4 % nucleotide difference from the Beilong virus we previously discovered (from *Rattus tanezumi* captured in 2014, GenBank: MN598981–MN598984) without obvious virus mutations. Overall, these data indicate that members of the genus *Jeilongvirus*, particularly Beilong virus, are the dominant paramyxoviruses circulating in rodents in Hubei Province and that their diversity is increasing. Notably, the paramyxoviruses identified in this study remain unclassified (at the species level) and may represent novel species, with the exception of Beilong virus.

Besides jeilongviruses, we also identified a novel henipavirus (Shiyan201) from *Crocidura tanakae* and a novel morbilli-like virus (Huangshi10) from *Niviventer confucianus*. These novel viruses show a sister relationship with other henipaviruses and morbilliviruses recognized by the ICTV and seem to fall into the novel subclades of rodent morbilliviruses and shrew and/or rodent henipaviruses reported in previous studies [18,53]. While many members of the genera *Henipavirus* and *Morbillivirus* can cause disease in humans and wildlife, the zoonotic and pathogenic potential of these novel viruses in their sister clade remain unknown. In 2012, Mojiang virus, a rat-borne henipavirus, was identified in Yunnan Province, southern China, after three mining workers contracted severe pneumonia without a known cause and died, but it is unclear whether Mojiang virus was responsible for these fatalities [54]. Recently, a novel henipavirus, named Langya henipavirus, was found in a febrile patient in eastern China, and was closely linked to Mojiang virus [28,54]. Subsequent investigation identified 35 patients with Langya henipavirus infection and suggested that the shrew may be a natural reservoir of Langya henipavirus [28], highlighting the risk of novel rodent-/shrew-borne henipaviruses. Notably, the novel henipavirus reported in this study shares 68.0 and 67.1 % nucleotide identity with Langya virus and Mojiang virus, respectively. Therefore, we aim to further monitor febrile patients in Shiyan city to determine whether this novel henipavirus could cause a public health disaster.

Interestingly, the broad diversity of paramyxoviruses observed in this study is also reflected in their genome structure. Although all paramyxoviruses share a similar genomic organization, 3′-N-P/V/C-M-F-RBP-L-5′, the six near-complete genomes we obtained have additional genes at different genomic locations, revealing the remarkable diversity in paramyxovirus genomic structures (Fig. 4). As described in previous studies [45], an interesting feature of jeilongviruses reported here is the presence of SH and/or TM genes between the F and G ORFs, as well as the G gene followed by an unknown ORF X (only found in Beilong virus, J-virus and Tailam virus currently). Of note, Jingzhou25 was closely related to J-virus, but ORF X was absent in its genome. Previous studies have reported that ORF X is not required for growth of Tailam virus in tissue culture cells and does not appear to be expressed in J-virus-infected cells [44,55]. This seems to indicate that the absence of ORF X may not affect the infection and replication of the virus. In addition, the unknown ORF was also found between the M and F ORFs of a novel henipavirus (Shiyan201) and between the F and G ORFs of a novel morbilli-like virus (Huangshi10). However, the function of the resulting protein, a small transmembrane protein, needs further study. The different genomic organizations described here suggest that the increased diversity of paramyxoviruses may be associated with their more complex genomic content except mutations of the genome.

Fig. 5

## supplementary material

10.1099/mgen.0.001229 Uncited Supplementary Material 1.

## Notes

F: fusion protein
HTS: high-throughput sequencing
HTS: high-throughput sequencing
ICTV: International Committee for the Taxonomy of Viruses
L: RNA polymerase
M: matrix protein
ML: maximum likelihood
mt: mitochondrial
N: nucleoprotein
P: phosphoprotein
RBD: receptor binding protein
RT-PCR: reverse transcription PCR
SH: small hydrophobic protein
TM: transmembrane protein
