# Supplementary material for: Discovery and genetic characterization of novel paramyxoviruses from small mammals in Hubei Province, Central China
Source: Microb Genom. 2024 May 3;10(5):001229. doi: 10.1099/mgen.0.001229 (PMC11145887; doi:10.1099/mgen.0.001229)
Supplement: Uncited Supplementary Material 1. [file mgen-10-01229-s001.pdf]

Huangshi Niviventer confucianus morbilli-like virus (Huangshi10)

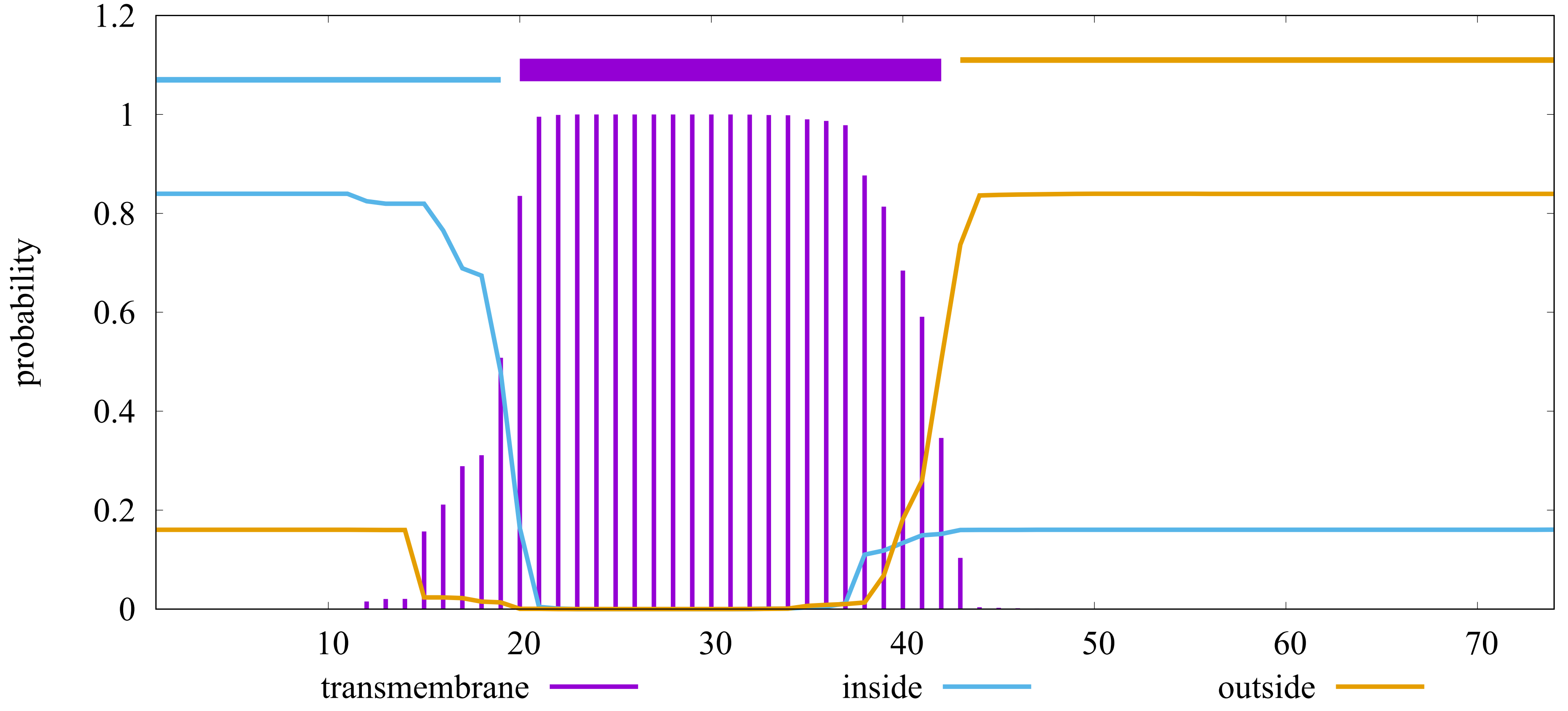

Shiyan Crocidura tanakae henipavirus (Shiyan201)

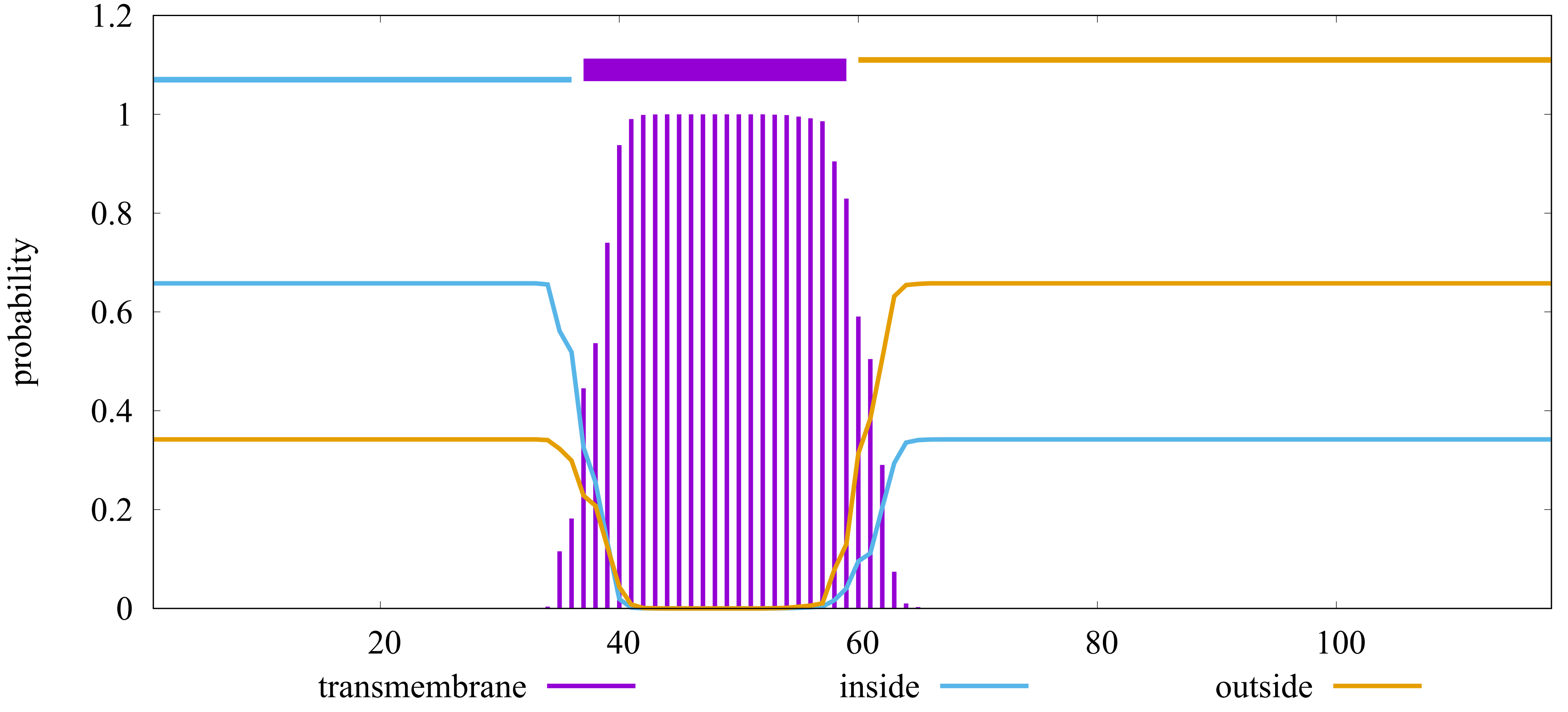

Figure S1: TMHMM posterior probabilities of ORF X Y and ORF Z in Huangshi Niviventer confucianus morbilli-like virus and Shiyan Crocidura tanakae henipavirus. The x-axis represents the amino acid length and the y-axis represents the posterior probability.

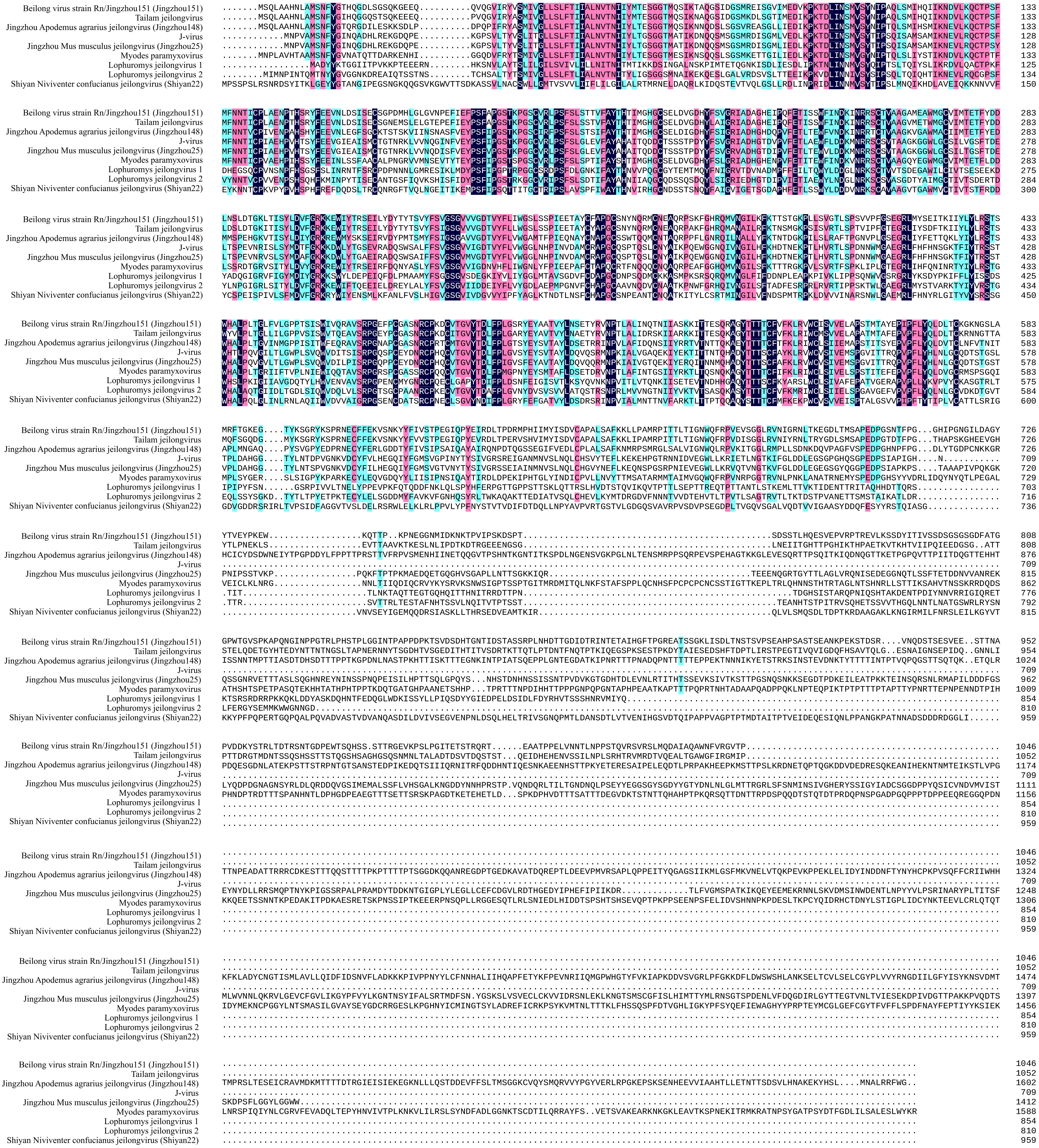

Figure S2: Multiple alignment of amino acid sequences of G proteins of jeilongviruses.

Table S1. Primers used for nested RT-PCR to obtain complete virus genome sequences.

Sample	First round		Second round	
	Primer name	Primer sequence (5’-3’)	Primer name	Primer sequence (5’-3’)
Huangshi10	1F	GCCTACCAACCATGTCAACA	N-1F	ATCAAACCACCTGATCCTTCTG
	1R	CTGACTACTCACTTGAATCCTAAG	N-1R	GTTCCAACTGCCATGAATCTC
	2F	AAGTCCCTTAGATGGTCTTGAT	N-2F	GAACACAACCAGGTGATTACTG
	2R	TCTCTTGACTTTCCCYCATATG	N-2R	CTTGTRGCATTAGTCTTGYTGTC
	2F	GGACGACCATATAGCTCTGTG	N-2F	CGATCTCAAACCTATTCTTGGA
	2R	CCARTGAACTTGAGCATTKGTG	N-2R	ACTAGATAATAGATCAGGAGCATG
	3F	GTCTACTGTGGAGGAACCAG	N-3F	CATCAGCTCCGAAAGAGTTC
	3R	GCTCTGATTGATCTAGCAGAAC	N-3R	GTTGAGTCTATCCATAGCATGC
	3F	GCATGCTATGGATAGACTCAAC	N-3F	GTTCTGCTAGATCAATCAGAGC
	3R	GCCACGAGCAATTTTAGCTG	N-3R	AAGGGTCTTCTTCTCCCATC
	4F	GATGGGAGAAGAAGACCCTT	N-4F	CAGCTAAAATTGCTCGTGGC
	4R	TGTTATYCCACAATCYTGYTGG	N-4R	GAAGAGCCCYTCTARTTTTGTAG
	4F	GACATAGGAAGAATACCCATTTCA	N-4F	GATTGCTACATTATCACAGTATGGA
	4R	AATCTYCCWACRCCGTTAGATAT	N-4R	CATGCTCTCATYTTRTAGGTCATC
	5F	ACTATCAATCCAACTATGACAGC	N-5F	AGACCTATACTTGACAATCCACA
	5R	GCATAGATAACCTTGCTGCTTC	N-5R	TTTCTGGAATTGAACGAAGGC
	5F	TCGYGACAARAGCACTCAAGT	N-5F	GGGAGRMGAGTRGACACYAAC
	5R	ATGGATTGAACTCTCGCTGAC	N-5R	ACCTAAGTAATGTTGATAGACTGC
	6F	TYGGTRTMTCACTCAACAATTACG	N-6F	ACTCAACTGCWTGTTAYAARGCT
	6R	CAAAATTGGCCATAGAAACTTACT	N-6R	TCAGGGACTGGAAGTGGAC
Shiyan201	1F	ACGTAATCCTTAAGAATCAAAGAT	N-1F	GTTCAGGGAGTATCAAGCAAAC
	1R	TGCCATCATYTGACCRAGTCTG	N-1R	CCATRGCRAARCTCCAWAGYAA
	2F	CAGTGAGAAAGTTCATGGTCGA	N-2F	GAAGTCAAGAAGAGCGGAGC
	2R	CYAACATCAGWGCYTTGTACTCC	N-2R	CCTCAGGTACAGAGTTGATGAAC
	2F	GAGGCGATAAGTGAAAGGCAT	N-3F	CATGACGACAAGCTCAAACAAAT
	2R	TTAGTCATRTCAAGTGGCTCCA	N-3R	AGGKCCTYTCATTTCWGATGACA
	3F	GATACAAGGAACAGAGTAGATGC	N-4F	CGTAGTGTAGGGAAATTAGGTCT
	3R	CWACWGCTCCYAAKGAGAAGAC	N-4R	CCTATATGAAGCATRAAYGTGGC
	3F	GATTTCAGAGAACACAATGCA	N-5F	CAATCTGCTTGTCTCTTTGCT
	3R	AKAYCATYAAWCCTCTRATCARTC	N-5R	TRCAACTRTTCTGCATYTGGWT
	4F	GATCAACCCGGAACAAATACCT	N-6F	TGTCAAGATAAGCACCTACCTTG
	4R	GGTGTWGCATARTCGTTGTAGC	N-6R	TCAAGAGAACGTATCGAGGGAC
	4F	CACTGGCAATATAATAGGTGTGG	N-7F	GCCCTGGAGATACATTTTCCTA
	4R	TGRCTKGGTATGGTKACACTRAC	N-7R	TTCGCCTTTRACAATTTGRTCTA
	5F	TTGCTGATGGGGTTACTAAT	N-8F	GGCTAACATGCTCCTAATAATCA
	5R	AAGCACTTAGTTATAGTTGAYCC	N-8R	CTGRTCTATRACCTCATTYGGTG
	5F	GGTAYGGWCARACAGARAGGA	N-9F	GYARTGGWGGTMGWATATGYCC
	5R	GTTRCAYTCKGGRTAYGGTACATG	N-9R	GACCYAATTTCCCATTYTGTATTC
	6F	TCRGATGTGYTKTATCCWGAATG	N-10F	TRGAYAGTCCTATWGTWGCWGG
	6R	ACTCTGGATCATTCTTATARTCACC	N-10R	TTCTGAACARATAGGTGATAATGC
	6F	GRGCWCATTCTAATGGYGAAGC	N-11F	CATGARATGTGTGCWACYAAYTGG
	6R	CCTARWGCWGCYARATTDTCYCT	N-11R	GWGCYCKYTCWGCACTRATTCT
	7F	GCCMTTRATACARAATCARTCATGG	N-12F	GCATGTCTCTTACCTGCACC
	7R	GRTGWAGATCMGWTATAGTCCATTC	N-12R	GRTCACTTTCKATCAAWGGRTTRC
	7F	TTTCARCARGTKATGCTCTTAGG	N-13F	CACCCAAGGARAATATCATAATGC
	7R	CATTGGYTCYTTMYCMATKCCAC	N-13R	CCAKGTTGTTTCWGGTCTSCC
	8F	GGATTTACTCCCATACATACTCC	N-14F	GACAGGGTATTAGGTCCAAGC
	8R	AACTCACTTGTCTGACTAGATC	N-14R	TACTTGGTTTTGGGACTGGC
